# Study of the Variability of Transcutaneous Bilirubin Determinations Between Different Ethnic Groups

**DOI:** 10.3390/children12050643

**Published:** 2025-05-16

**Authors:** Laia Plaza, Neus Roca Saladrigues, Meritxell Torrabías, Fina Bueno, Marina Damas, Carmina Parès, Jacint Altimiras, Marta Rodríguez González

**Affiliations:** 1 Faculty of Medicine, Universitat de Vic-Universitat Central de Catalunya, 08500 Vic, Spain; 2 Pediatric Department, Consorci Hospitalari de Vic, 08500 Vic, Spain; nroca@chv.cat (N.R.S.); mrodriguezg@chv.cat (M.R.G.); 3 Clinical Epidemiology Department, Hospital Universitari de Vic, 08500 Vic, Spain

**Keywords:** neonatal hyperbilirubinemia, neonatal jaundice, kernicterus, phototherapy, ethnicity, skin pigmentation

## Abstract

Background: Pathological hyperbilirubinemia often leads to hospital readmission within the first week of life, with increased risk of neurological damage if untreated. Transcutaneous bilirubin (TcB) measurement was integrated into neonatal screening to estimate total serum bilirubin (SB) concentrations. Despite TcB and SB generally correlating well, discrepancies can occur based on race/ethnicity. Falsely elevated TcB readings may be obtained in darker skin pigmentation. Aims: This study compared TcB and SB across different ethnic groups to assess correlation patterns and identify the best TcB measurement method in neonates. Methods: Term and late preterm neonates delivered at the University Hospital of Vic were included. Each newborn underwent TcB assessment (in the forehead, sternum, and both sites simultaneously) concomitantly with SB measurement. The correlations between both parameters were analyzed. Results: A total of 148 neonates were categorized as White/Caucasian (58), Chinese (3), Indian (17), Black/African (22), Latino (11), Arab (25), or mixed (12). The groups were homogeneous, with statistical differences in delivery and feeding (*p* = 0.032 and *p* < 0.001). Differences between TcB and SB were −0.19 for White/Caucasian, 0.90 for Chinese, 1.12 for Indian, 2.47 for Black/African, 0.42 for Latino, and −0.08 for Arab (*p* < 0.001). A high association between TcB and SB was obtained with all measurement methods: r = 0.836 in forehead, r = 0.869 in midsternum, and r = 0.863 when both locations were combined (*p* < 0.001). Conclusions: TcB correlated well with SB, but accuracy varied among ethnic groups. An individualized interpretation of TcB based on skin pigmentation is supported. Mid-sternum determination was the best TcB measurement method.

## 1. Introduction

More than 80% of newborns will have some degree of jaundice, caused by a product of hemoglobin catabolism called bilirubin [1]. Physiological jaundice is a frequent situation in term neonates [2,3], becoming pathological when it starts in the first 24 h, and it is accompanied by other symptoms when bilirubin exceeds the defined limits, bilirubin increases by more than 5 mg/dL daily, the direct fraction is greater than 2 mg/dL, or it lasts for more than two weeks (except if breastfeeding, in which case it may last for three weeks or longer) [2].

Pathological hyperbilirubinemia is one of the main reasons for hospital readmission in the first week of life [4,5]. The importance of close monitoring and the early detection of elevated bilirubin levels is due to the risk of neurological damage if appropriate treatments are not established. Although most newborns with hyperbilirubinemia have a good prognosis, 8–11% maintain high bilirubin levels, which may lead to complications [6]. High bilirubin concentrations can cause acute encephalopathy and kernicterus, a permanently disabling neurologic condition caused by the deposition of bilirubin in the brain [1,3,7]. The incidence of severe hyperbilirubinemia increases if risk factors are present [6,8,9]. G6PD deficiency, ABO incompatibility, premature birth, birth trauma (e.g., bruising or cephalohematoma), male sex, and Asian ethnicity are risk factors for pathological neonatal hyperbilirubinemia [6]. These risk factors are assumed to vary between populations.

Visual estimation of bilirubin levels according to the degree of jaundice and its cephalocaudal progression can lead to errors [5,10]. In order to improve detection, the measurement of transcutaneous bilirubin (TcB) has been introduced into neonatal screening prior to hospital discharge [5]. A transcutaneous bilirubinometer evaluates the yellowness of the skin. The device emits light of different wavelengths and collects the reflectance from the skin surface, which depends on the concentration of different skin chromophores, including bilirubin. Each chromophore has a different absorption spectrum, enabling the calculation of its concentration. In this way, the concentration of TcB allows for the estimation of the total serum bilirubin (SB) concentration non-invasively from the objective measurement of skin color [1,4,11].

In the case of obtaining high TcB values, it is necessary to determine SB invasively to verify it. The available literature demonstrates good correlation between SB concentration and TcB measurements obtained using BiliCheck and JM instruments. Therefore, transcutaneous bilirubinometry is considered a valid screening method for neonatal hyperbilirubinemia, identifying who requires a confirmatory SB measurement [1,8,12]. Accordingly, using TcB measurements may result in a reduction in blood draws [1]. Although these devices seem accurate for measuring TcB, underestimation and overestimation occur based on the SB concentration, measurement site, and race/ethnicity [8,13]. The magnitude and direction of the average difference between TcB measurements and SB concentrations may depend on skin’s melanin concentration and the device used [10,14]. Although previous studies have suggested that TcB readings may be influenced by skin pigmentation, the exact amount of difference between ethnic groups has not been described yet, nor has the reliability of different measurement sites been evaluated.

In clinical practice, with the Draëger JM-105 device, falsely higher TcB values are obtained in infants with increased skin pigmentation, which do not correlate with SB levels [13]. In addition, the exact variability between different ethnicities according to their pigmentation degree is not specified.

The nomograms currently used in clinical practice are those recommended by the SENEO (Spanish Society of Neonatology) from the latest updates of the AAP (American Academy of Pediatrics) in 2022. Genetic background, along with biological and epidemiological differences in the natural history of neonatal hyperbilirubinemia, have led to the development of population-specific nomograms in several countries [13]. However, TcB concentrations, increase rates, and peaks vary among ethnicities, highlighting the specific natural course of jaundice in different parts of the world [8,15,16]. The nomogram used should reflect the population that most closely represents the neonate under evaluation [8,13].

In order to increase the reliability of hour-specific nomograms, previous studies have recognized the need for significant research to evaluate the role of race/ethnicity [8].

The main hypothesis of the project supports that in the non-Caucasian race (especially in skin with higher pigmentation), there is no good correlation between the concentration of TcB and SB.

The aim of the study is to compare TcB values with SB values in term neonates to assess whether there is a correlation pattern determined by different ethnic groups and, consequently, if TcB should be interpreted distinctively according to ethnicity so that it predicts SB better.

As a secondary objective, it is intended to assess whether TcB levels vary according to the place of measurement (three determinations in the forehead, three determinations in the sternum, or a determination made in both places simultaneously [forehead–sternum–forehead]).

## 2. Materials and Methods

### 2.1. Sample Selection Process

The design of the present study was prospectively observational. The participants eligible to be included in the study were all neonates with ≥35 weeks of gestation born at the University Hospital of Vic whose parents signed the informed consent. Patient recruitment took place over 4 months (from 11 December 2023 to 8 April 2024). The sample participating in the study was representative of the ethnic diversity of the local births. The exclusion criteria included neonates who were receiving phototherapy treatment at the time of sampling, those who had received phototherapy less than 24 h ago, or if they had signs of active haemolysis (because TcB may have underestimated SB).

This study was previously approved by an Ethics Committee and it was conducted in accordance with the Helsinki Declaration.

### 2.2. Data Extraction

Epidemiological and clinical data on the follow-up of pregnancy, childbirth, and the first hours of life of the newborn were collected anonymously. The variables collected were divided into clinical (maternal age, maternal and paternal ethnicities, maternal blood group, indirect Coombs, family history of G6PD deficiency, family history of jaundice or need for phototherapy, history of gestational diabetes, mode of delivery, gestational age, sex of the newborn, birth weight, neonatal blood group, direct Coombs, G6PD deficiency in the neonate, major congenital abnormalities, birthmarks on the forehead or sternum, Down syndrome, cephalohematoma, septic signs, feeding method, hours of life at the onset of lactation, presence of ≥10% loss of birth weight, presence of clinical jaundice and hours of life at detection, need for phototherapy and hours of life at the onset, duration of phototherapy, need for immunoglobulins or exsanguinotransfusion, and the presence of associated hemolytic anemia) and analytical variables (determination of TcB in the forehead, determination of TcB in the sternum, determination of TcB in the forehead and the sternum combined, and the determination of SB). The ethnicity of the newborn was determined according to the ethnicity of both parents, subclassified as White/Caucasian, Chinese, Indian, Black/African, Latino, Arab, or mixed ethnicity.

Prior to discharge, each participating newborn was tested for TcB, which is part of routine neonatal screening. To measure TcB, an average of 3 determinations were made, obtained via direct contact of the skin with the calibrated bilirubinometer at the same point (forehead, sternum, or a combination of forehead and sternum). The transcutaneous bilirubinometer used for the study was the JM-105 (Draëger Medical Systems, Inc., Telford, PA, USA). At the same time, a blood sample was taken to determine SB accurately. The analytical method used to assess SB was colorimetric diazo. In case the newborn needed to be observed until 48 h of life, the same puncture of the routine heel test was used to take the SB sample.

Two separate databases were created for the study. In the first one, an identification number was assigned to the set of medical records of the mother and her newborn. In this way, each enrolled neonate was registered as a unique identifier, which allowed its anonymity to be preserved. In the second one, the data obtained were already recorded and anonymized.

### 2.3. Statistical Analysis

The estimated sample size in order to be able to compare the different ethnic subgroups and obtain significant results was around 140 participants.

For the statistical analysis plan, a univariate statistical analysis of all variables was performed. Qualitative data were expressed in absolute and relative frequencies; and quantitative data in means, medians, typical deviations, and percentiles (25th and 75th percentiles). There was also a bivariate statistical analysis between the variables of TcB and SB, taking into account the total sample (Pearson’s correlation test) and then repeated by ethnic groups (Pearson’s or Spearman’s correlation tests). Furthermore, the difference between SB and the mean determination of TcB measurements was compared among the ethnicities included (ANOVA test). In the case of small samples, or where the conditions for the application of these statistical tests did not exist, non-parametric statistics were used. The significance level considered was 5%. All tests of statistical significance were two-tailed at a 95% confidence interval. The statistical program used was the IBM SPSS Statistics version 29.0.2.0.

## 3. Results

**Figure 1:**
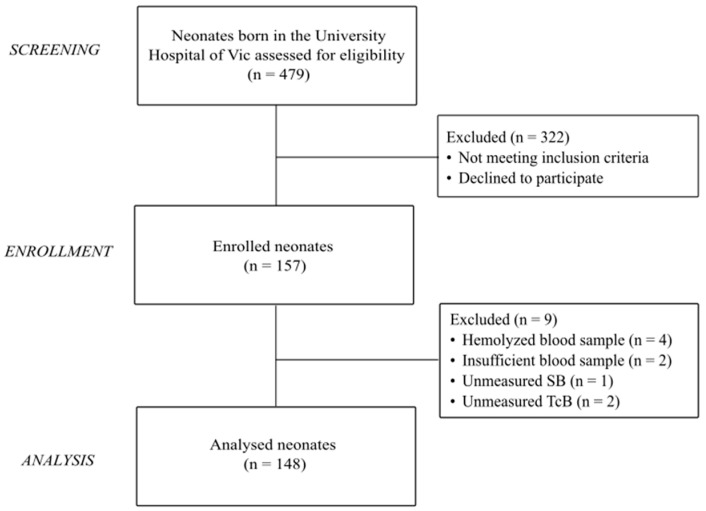
Flowchart of participants. SB: serum bilirubin; TcB: transcutaneous bilirubin.

During the recruitment period, 157 neonates met the selection criteria and were enrolled in the study. Of them, nine infants were excluded from the record for missing data of SB or TcB readings or due to problems in the processing of the blood sample. The final study cohort eligible for analysis consisted of 148 newborns (Figure 1).

Table 1 gives demographic details of the population enrolled in the study. The sample analyzed consisted of 148 neonates, from which paired measurements of TcB and SB were collected. The study population was representative of the ethnic diversity of the inner city population, classified as White/Caucasian [58 (39.2%)], Chinese [3 (2%)], Indian [17 (11.5%)], Black/African [22 (14.9%)], Latino [11 (7.4%)], Arab [25 (16.9%)], or mixed [12 (8.1%)] according to the ethnicity of both parents. Mixed ethnicity was considered to be those newborns who had two different paternal ethnicities.

No significant differences were observed in gestational age and sex between ethnicities. The mode of delivery and feeding method differed significantly among ethnicities (*p* = 0.032 and *p* < 0.001, respectively). No significant differences were observed in jaundice risk factors across the populations.

The TcB measurements tested in the study were defined as determination in the forehead, determination in the sternum, and determination in the forehead–sternum–forehead. Some of the infants had repeated TcB measurements. However, only the first determinations were considered for analysis. SB level measurement was also performed.

Of the 148 paired measurements taken, the median average of the three TcB determinations was 9.9 mg/dL, with an interquartile range from 7.2 mg/dL to 12.1 mg/dL. The median SB was 9.4 mg/dL, with an interquartile range from 7.4 mg/dL to 11.8 mg/dL.

**Figure A1:**
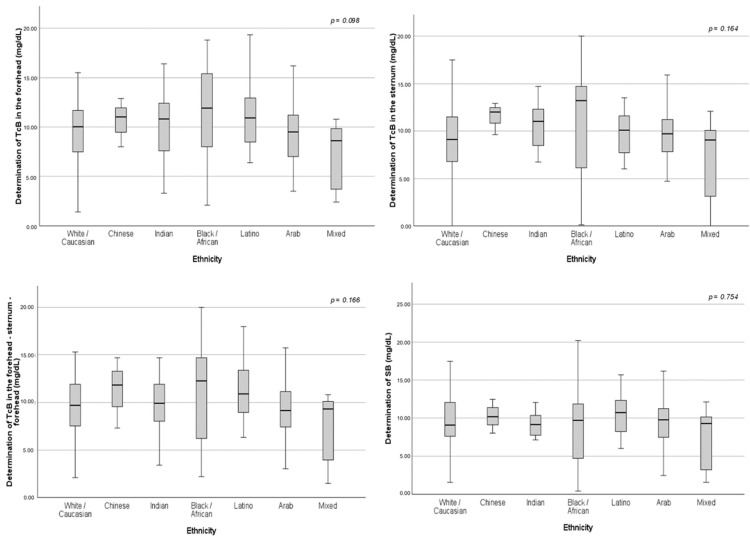
Box plots showing median levels of the baseline bilirubin determinations—comparison between the different ethnic groups. SB: serum bilirubin; TcB: transcutaneous bilirubin.

The mean TcB values were higher for the Black/African population. Meanwhile, the SB levels were greater for the Latino group. However, there were no significant differences among the different ethnicities (Table 2 and Figure A1).

**Figure 2:**
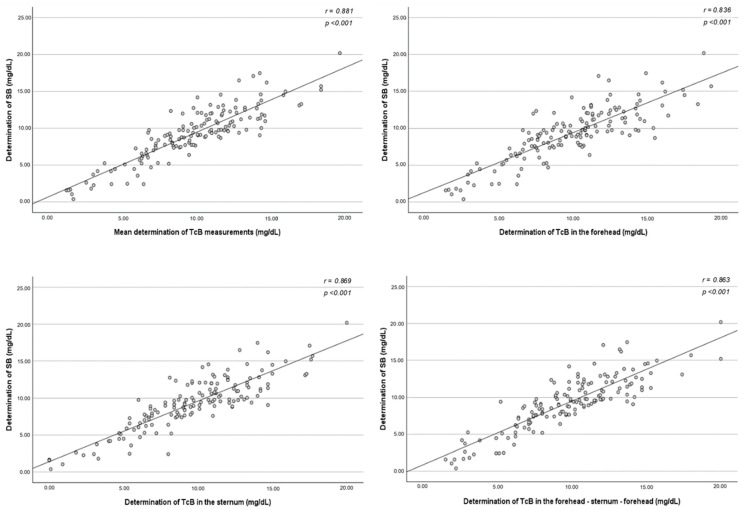
Correlation between the different TcB determinations and the determination of SB—analysis of the global sample of newborns. SB: serum bilirubin; TcB: transcutaneous bilirubin.

Figure 2 shows the relationship between each method of TcB measurement and the SB determination in reference to the line of identity (45°) for all measurements. The correlation between SB concentration and the mean of the three TcB determinations was also studied. Based on this model, the TcB levels seemed to have good correlation with the SB concentration regardless of the method used for determination. When comparing all 148 paired measurements, an especially high association was obtained when the TcB levels were tested in the mid-sternum, where the Pearson’s correlation coefficient was r = 0.869, 95% CI: lower limit to upper limit (*p* < 0.001).

The same correlation analysis was conducted and segmented by ethnicity. In the ethnicity-segmented analysis, the group of mixed-race newborns was not included due to its heterogeneity.

**Figure A2:**
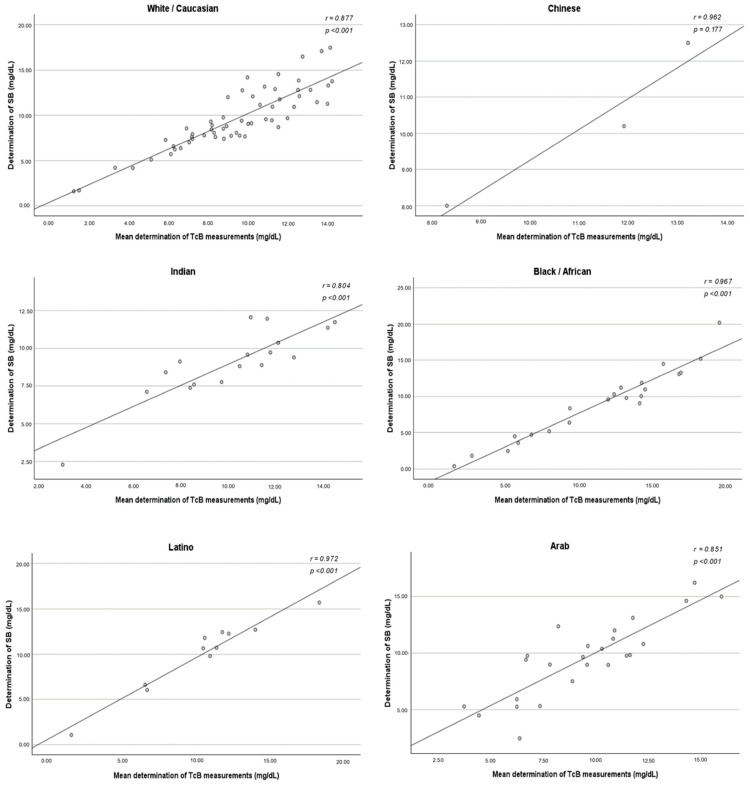
Correlation between the mean determination of the TcB measurements and the determination of SB—ethnicity-segmented analysis. SB: serum bilirubin; TcB: transcutaneous bilirubin.

**Figure A3:**
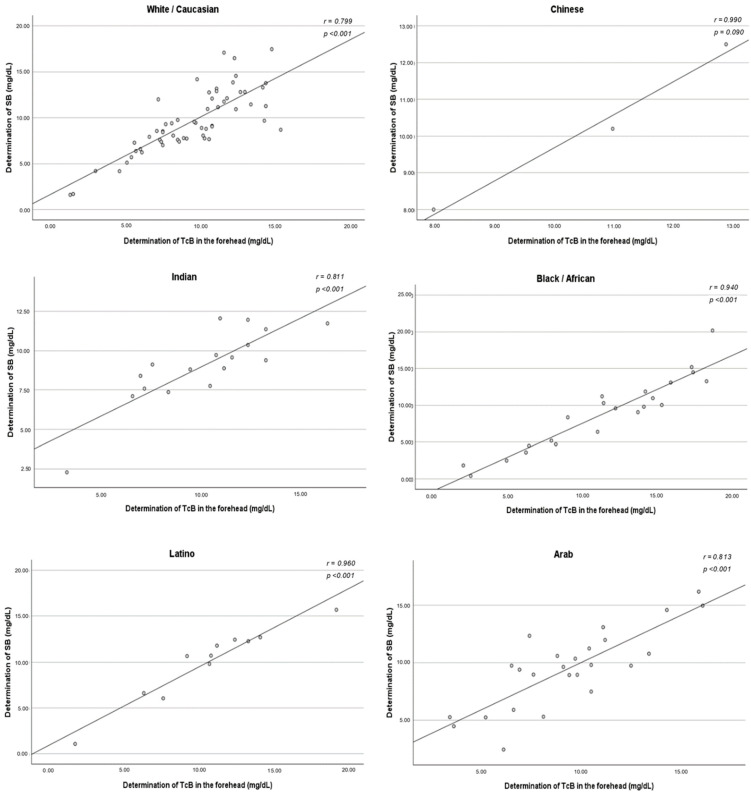
Correlation between the determination of TcB in the forehead and the determination of SB—ethnicity-segmented analysis. SB: serum bilirubin; TcB: transcutaneous bilirubin.

**Figure A4:**
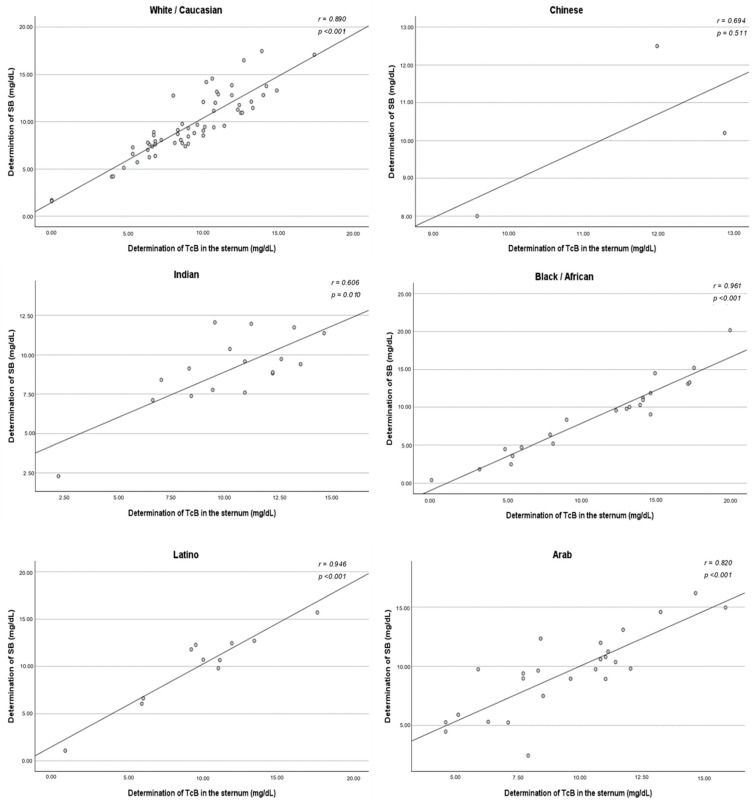
Correlation between the determination of TcB in the sternum and the determination of SB—ethnicity-segmented analysis. SB: serum bilirubin; TcB: transcutaneous bilirubin.

**Figure A5:**
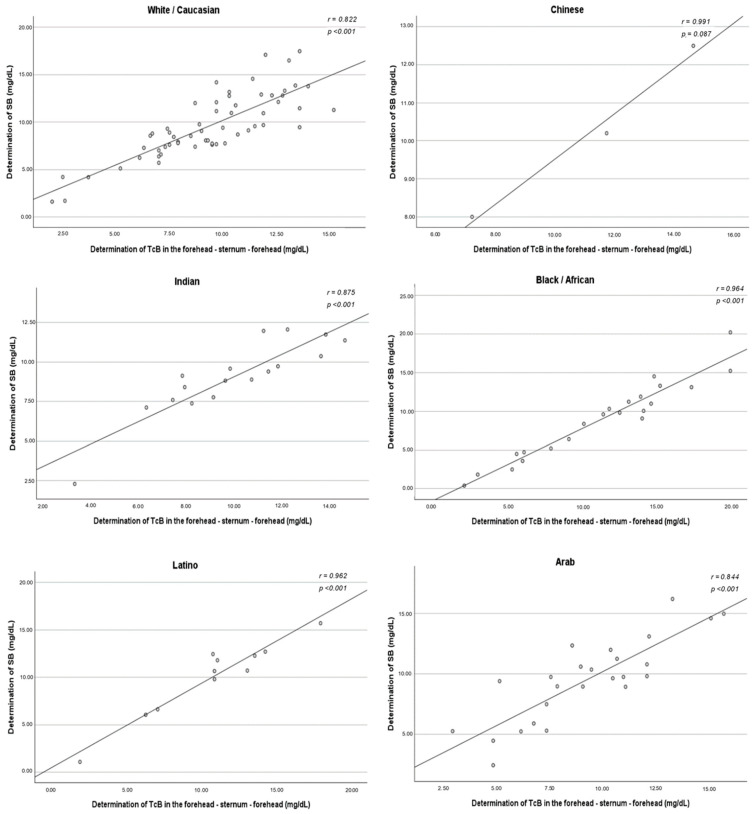
Correlation between the determination of TcB in the forehead–sternum–forehead and the determination of SB—ethnicity-segmented analysis. SB: serum bilirubin; TcB: transcutaneous bilirubin.

The correlation between TcB and SB did not vary among ethnic groups. A strong positive association was found in all ethnic subgroups, with statistical significance obtained except for Chinese newborns. The method of measuring TcB that best correlated with the SB concentration varied according to the ethnicity studied. For White/Caucasians, the method that demonstrated the strongest correlation was the three determinations taken in the sternum (r = 0.890). However, the method of measuring in the forehead and sternum simultaneously demonstrated a stronger correlation in the Chinese (r = 0.991), Indian (r = 0.875), Black/African (r = 0.964), Latino (r = 0.962), and Arab (r = 0.844) ethnicities (Figure A2, Figure A3, Figure A4 and Figure A5).

The difference between the determination of the SB concentration and the mean of the three TcB measurements was calculated for each ethnic group enrolled. A significant difference was found in the average variation between the SB determination and the mean TcB determination depending on the ethnic group (*p* < 0.001).

Moreover, the resulting average difference was either positive or negative, indicating an overestimation or underestimation of the SB values from the mean TcB levels. On average, the TcB values tended to overestimate the SB values. In White/Caucasians, the mean TcB was 0.19 points lower than the SB. In Asians, the mean TcB was 0.90 points higher than the SB. In Indians, the mean TcB was 1.12 points higher than the SB. In Black/Africans, the mean TcB was 2.47 points higher than the SB. In Latinos, the mean TcB was 0.42 points higher than the SB. In Arabs, the mean TcB was 0.08 points lower than the SB (Table 3).

A final Bonferroni analysis of multiple comparisons was performed to know specifically among which ethnic subgroups the significant differences were found in Table 3. The significant differences were found between White/Caucasian and Indian ethnicities (*p* = 0.048); White/Caucasian and Black/African ethnicities (*p* < 0.001); Black/African and Latino ethnicities (*p* = 0.008); and Black/African and Arab ethnicities (*p* < 0.001) (Table A1).

## 4. Discussion

The reviewed literature highlights the importance of introducing transcutaneous bilirubinometry in clinical practice as a substantial advancement in the non-invasive detection of neonatal hyperbilirubinemia [1,5,17]. Nevertheless, the accuracy of TcB measurements may be influenced by a variety of factors, including skin melanin concentration. This fact is particularly pertinent considering the acknowledged variations in skin pigmentation distribution among different ethnic groups. Other preceding studies have developed TcB nomograms for different populations, but no comparisons have been analyzed in a multi-ethnic population [8,13,15,16]. This research project relied on an ethnically diverse cohort of newborns to address the issue of the accuracy of the transcutaneous device among newborns with various degrees of skin pigmentation.

The present study aimed to investigate the correlation between transcutaneous bilirubin (TcB) levels and total serum bilirubin (SB) levels in neonates from various ethnic groups. The study addressed this question by comparing the TcB and SB levels in neonates from different ethnic groups. In the same way as in previous reports, the results of our study indicated a good correlation between the levels of TcB and SB in the population studied as a whole [4,12]. However, validated bilirubinometers allow for a degree of error (approximately 3 mg/dL), so it is always required to perform the determination of SB prior to the decision to start treatment with phototherapy, according to the graphs on hours of life and gestational age of the American protocols.

The main objective was to determine whether an ethnicity-dependent correlation pattern existed and could modify the interpretation of TcB to improve the prediction of SB levels. Variations in the accuracy of TcB were observed according to the ethnic group, with some populations showing an overestimation, while others showed an underestimation of the SB levels based on TcB measurements. Confirming the findings previously reported by Olusanya et al., it was observed that neonates of African and Indian descent, and therefore with higher skin pigmentation, had a significant overestimation of TcB levels compared to the rest of the ethnic populations [13]. The differences obtained highlight the importance of an individualized interpretation of TcB measurements considering the ethnicity of the neonate. This is especially relevant in the case of high bilirubin levels, which are commonly seen in the first week of life. The variations found in the bilirubin levels could be due, in part, to well-known genetic differences typical of each race/ethnicity. As summarized in Table 1, slight dissimilarities in some jaundice risk factors may also explain some of the variations.

Furthermore, it was also evaluated whether TcB levels vary depending on the measurement site in the body of the newborn. The user manuals of the validated bilirubinometers do not specify the most optimal place to take the three TcB measurements, recommending that it be on the forehead or on the sternum. For this reason, different centers and even different professionals in the same center take the measurement indistinctly on the forehead, the sternum, or alternating between both locations. The TcB levels seemed to have a good correlation with the SB concentration regardless of the method used for their determination. It appears that the site where the TcB was measured with the transcutaneous device (forehead or sternum) did not influence a better or worse estimate of the SB concentration. Despite this, sampling in the sternum was the one with the best correlation. These findings could be due, as Kuboi et al. mentioned in their report, to the fact that measurements from the mid-sternum are probably a better choice, because the forehead is exposed to ambient light while the sternum is almost always covered [16]. This may be explained by the same factor in other investigations, which concluded that TcB devices are much less accurate in estimating SB values in infants when receiving phototherapy compared to their documented accuracy in the pre-phototherapy period [18,19]. Nevertheless, it cannot be recommended to use a particular method over another without an appropriate cost-effectiveness analysis.

Some limitations of the present analysis should be mentioned, such as the small size of the sample compared with other reports and the representativeness of the ethnic populations studied. Although it was a representative population sample of the local births, there appeared to be an unequal ethnicity distribution in the sample, with a majority of White/Caucasians. Ethnic minorities had smaller numbers in the sample, limiting statistical robustness. Therefore, caution must be used when extrapolating our findings to Chinese neonates, because they represent the smallest proportion of the studied populations, and no conclusions regarding this group can reliably be made. Moreover, infant ethnicity may not be the best indicator of skin pigmentation. Certain biases would probably have been avoided by considering skin phototype rather than ethnicity as a classification criterion. Future research should consider objective scales such as the melanin index or the Fitzpatrick classification to precisely reflect the newborn’s actual skin color. The classification method for mixed-ethnicity individuals was not initially considered, which turned out to be a group too heterogeneous to be included in the statistical analysis. In this way, attempts were made to avoid possible biases in the results.

The data obtained suggest that additional research is needed to validate these findings in different clinical contexts and ethnic populations. Future studies should consider performing multivariate regression analyses in order to better isolate the effect of ethnicity on TcB accuracy, while also controlling for relevant clinical factors such as ABO incompatibility, G6PD deficiency, type of feeding, and gestational age. Although bilirubin measurements were taken prior to discharge, slight variations in age at the time of measurement may affect the absolute values. Future studies should consider adjusting for postnatal age when comparing bilirubin levels. The findings from this single hospital would require further validation in a multi-center study, including a larger sample size for each ethnic subgroup with equal representativeness in order to facilitate satisfactory generalization at the population level. Given our results, it would be good to be able to be more precise in the interpretation of TcB in the different ethnic groups, either by considering subtracting or adding points to the value obtained according to race or by creating more detailed bilirubinometers that take into account skin phototype and can solve the bias of the current devices. These findings have significant implications for clinical practice, particularly in settings where ethnic diversity is high due to the increase in migratory flows. Incorporating ethnic considerations into the interpretation of TcB measurements could improve the accuracy of diagnosis and the management of neonatal hyperbilirubinemia, which could potentially reduce the risk of associated complications.

## 5. Conclusions

In the present investigation of ethnically diverse late preterm and term newborns, the JM-105 device correlated but was often imprecise in predicting actual SB levels according to the ethnicity studied. The conclusions of the study support the main hypothesis by demonstrating that the correlation between the TcB and SB concentrations can vary across different ethnic groups. While a good correlation between TcB and SB was observed in the population studied as a whole, it was found that this correlation may be less accurate in non-Caucasian ethnic groups, particularly in neonates with higher skin pigmentation, such as Africans and Indians, where a greater discrepancy between the TcB and SB levels was obtained. Therefore, the interpretation of TcB measurements should take into account the ethnicity of the newborn to improve the prediction of SB levels.

Aligned with the secondary objective of the study, it was found that there are no considerable differences between the methods of measuring TcB (three determinations in the forehead, in the sternum, or in both places simultaneously), as they all had a fairly high correlation coefficient (r). The three determinations of the sternum stood out as the ones with the strongest association with SB.

In summary, this study provides evidence supporting the importance of considering ethnic differences and the place of measurement when interpreting TcB determinations in neonates, which could improve diagnostic accuracy and the management of neonatal hyperbilirubinemia. However, more research to validate these findings and further explore the impact of ethnicity and measurement method on the accuracy of TcB is required. Future studies should aim for larger and more balanced samples across all ethnic groups.

## Figures and Tables

**Table 1:** Baseline characteristics of the sample included in the study—comparison between the different ethnic groups.

	Total *n* = 148	White/Caucasian *n* = 58	Chinese *n* = 3	Indian *n* = 17	Black/African *n* = 22	Latino *n* = 11	Arab *n* = 25	Mixed Ethnicity *n* = 12	*p*-Value
**Gestational age (weeks), *median [IQR]***	39.3 [38.3–40.4]	39.3 [38.4–40.2]	38.6 [35.5–39.4]	39.5 [38.4–40.8]	39.4 [38.1–40.6]	40.2 [39.3–41.0]	39.3 [38.2–40.4]	39.1 [37.7–40.5]	0.518 ^a^
**Sex, *n (%)***									0.286 ^b^
Male	80 (54.1)	33 (56.9)	1 (33.3)	10 (58.8)	14 (63.6)	2 (18.2)	14 (56.0)	6 (50.0)	
Female	68 (45.9)	25 (43.1)	2 (66.7)	7 (41.2)	8 (36.4)	9 (81.8)	11 (44.0)	6 (50.0)	
**Mode of delivery, *n (%)***									**0.032** ^b^
Eutocic	91 (61.5)	38 (65.5)	3 (100.0)	13 (76.5)	13 (59.1)	1 (9.1)	15 (60.0)	8 (66.7)	
Cesarean section	47 (31.8)	16 (27.6)	0 (0.0)	4 (23.5)	8 (36.4)	9 (81.8)	6 (24.0)	4 (33.3)	
Instrumental	10 (6.8)	4 (6.9)	0 (0.0)	0 (0.0)	1 (4.5)	1 (9.1)	4 (16.0)	0 (0.0)	
**Birth weight, *n (%)***									0.314 ^b^
Underweight (<−2 SD)	3 (2.0)	1 (1.7)	0 (0.0)	0 (0.0)	1 (4.5)	0 (0.0)	0 (0.0)	1 (8.3)	
Normal weight	139 (93.9)	55 (94.8)	3 (100.0)	17 (100.0)	21 (95.5)	9 (81.8)	23 (92.0)	11 (91.7)	
Macrosomia (>2 SD)	6 (4.1)	2 (3.4)	0 (0.0)	0 (0.0)	0 (0.0)	2 (18.2)	2 (8.0)	0 (0.0)	
**Direct Coombs, *n (%)***									0.262 ^b^
Positive	3 (2.0)	3 (5.2)	0 (0.0)	0 (0.0)	0 (0.0)	0 (0.0)	0 (0.0)	0 (0.0)	
Negative	31 (20.9)	16 (27.6)	2 (66.7)	3 (17.6)	5 (22.7)	1 (9.1)	3 (12.0)	1 (8.3)	
Not tested	114 (77.0)	39 (67.2)	1 (33.3)	14 (82.4)	17 (77.3)	10 (90.9)	22 (88.0)	11 (91.7)	
**Feeding method, *n (%)***									**<0.001** ^b^
Breastfeeding	108 (73.0)	43 (74.1)	0 (0.0)	14 (82.3)	18 (81.8)	7 (63.6)	18 (72.0)	8 (66.7)	
Formula	11 (7.4)	6 (10.3)	3 (100.0)	0 (0.0)	0 (0.0)	0 (0.0)	1 (4.0)	1 (8.3)	
Mixed	29 (19.6)	9 (15.5)	0 (0.0)	3 (17.6)	4 (18.2)	4 (36.4)	6 (24.0)	3 (25.0)	
**≥10% loss of birth weight, *n (%)***									0.137 ^b^
No	133 (89.9)	51 (87.9)	3 (100.0)	16 (94.1)	21 (95.5)	11 (100.0)	19 (76.0)	12 (100.0)	
Yes	15 (10.1)	7 (12.1)	0 (0.0)	1 (5.9)	1 (4.5)	0 (0.0)	6 (24.0)	0 (0.0)	
**Family history of jaundice and need for phototherapy, *n (%)***									0.505 ^b^
No	144 (97.3)	56 (96.6)	3 (100.0)	17 (100.0)	22 (100.0)	11 (100.0)	23 (92.0)	12 (100.0)	
Yes	4 (2.8)	2 (3.4)	0 (0.0)	0 (0.0)	0 (0.0)	0 (0.0)	2 (8.0)	0 (0.0)	
**Presence of clinical jaundice, *n (%)***									0.751 ^b^
No	81 (54.7)	31 (53.4)	1 (33.3)	9 (52.9)	14 (63.6)	4 (36.4)	14 (56.0)	8 (66.7)	
Yes	67 (45.3)	27 (46.6)	2 (66.7)	8 (47.1)	8 (36.4)	7 (63.6)	11 (44.0)	4 (33.3)	
**Required phototherapy, *n (%)***									0.464 ^b^
No	135 (91.2)	52 (89.7)	3 (100.0)	17 (100.0)	18 (81.8)	11 (100.0)	23 (92.0)	11 (91.7)	
Yes	13 (8.8)	6 (10.3)	0 (0.0)	0 (0.0)	4 (18.2)	0 (0.0)	2 (8.0)	1 (8.3)	

Data presented as *n* (%) or median [25th percentile–75th percentile]. ^a^ Kruskal–Wallis. ^b^ Pearson χ^2^.

**Table 2:** Baseline bilirubin determinations—comparison between the different ethnic groups.

	Total *n* = 148	White/Caucasian *n* = 58	Chinese *n* = 3	Indian *n* = 17	Black/African *n* = 22	Latino *n* = 11	Arab *n* = 25	Mixed Ethnicity *n* = 12	*p*-Value
**Determination of TcB in the forehead (mg/dL), *median [IQR]***	10.3 [7.5–12.4]	10.1 [7.5–11.8]	11.0 [8.0–12.9]	10.8 [7.4–12.4]	11.9 [7.6–15.6]	10.9 [7.7–13.4]	9.5 [6.9–11.3]	8.6 [3.3–10.1]	0.098 ^a^
**Determination of TcB in the sternum (mg/dL), *median [IQR]***	9.7 [6.9–12.3]	9.1 [6.8–11.6]	12.0 [9.6–12.9]	11.0 [8.5–12.5]	13.2 [6.0–14.8]	10.1 [6.1–12.0]	9.7 [7.5–11.4]	9.1 [3.1–10.3]	0.164 ^a^
**Determination of TcB in the forehead–sternum–forehead (mg/dL), *median [IQR]***	9.8 [7.4–12.1]	9.7 [7.5–11.9]	11.8 [7.3–14.7]	9.9 [8.0–12.1]	12.3 [6.2–14.8]	10.9 [7.1–13.6]	9.1 [7.1–11.6]	9.3 [3.4–10.2]	0.166 ^a^
**Average of the three TcB determinations (mg/dL), *median [IQR]***	9.9 [7.2–12.1]	9.7 [7.2–11.6]	11.9 [8.3–13.2]	10.8 [8.2–12.0]	12.7 [6.6–14.9]	10.9 [6.7–12.2]	9.6 [6.7–11.5]	9.1 [3.3–10.0]	0.134 ^a^
**Determination of SB (mg/dL), *median [IQR]***	9.4 [7.4–11.8]	9.1 [7.6–12.1]	10.2 [8.0–12.5]	9.1 [7.7–10.9]	9.7 [4.7–12.2]	10.7 [6.6–12.4]	9.8 [6.7–11.6]	9.3 [2.9–10.1]	0.754 ^a^

SB: serum bilirubin; TcB: transcutaneous bilirubin. Data presented as median [25th percentile–75th percentile]. ^a^ Kruskal–Wallis.

**Table 3:** Difference between the mean determination of TcB measurements and the determination of SB—comparison between ethnic groups.

	Total *n* = 148	White/Caucasian *n* = 58	Chinese *n* = 3	Indian *n* = 17	Black/African *n* = 22	Latino *n* = 11	Arab *n* = 25	*p*-Value
**Difference between the mean determination of TcB and the determination of SB (mg/dL), *mean (SD)***	0.45 (1.8)	−0.19 (1.6)	0.90 (0.7)	1.12 (1.5)	2.47 (1.3)	0.42 (1.1)	−0.08 (1.8)	**<0.001** ^a^

SB: serum bilirubin; TcB: transcutaneous bilirubin. Data presented as mean (SD). ^a^ ANOVA.

## Data Availability

The original contributions presented in the study are included in the article, further inquiries can be directed to the corresponding authors.

## Abbreviations

The following abbreviations are used in this manuscript:

AAP	American Academy of Pediatrics
G6PD	Glucose-6-phosphate dehydrogenase
SB	Serum bilirubin
SENEO	Spanish Society of Neonatology
TcB	Transcutaneous bilirubin

## Appendix A

**Table A1:** Bonferroni test of multiple comparisons with the ‘difference between the determination of SB and the mean determination of TcB measurements’ as the dependent variable—comparison between ethnic groups.

		*p*-Value	95% Confidence Interval	
			Lower Limit	Upper Limit
White/Caucasian	Chinese	1.000	−3.88	1.71
	Indian	**0.048**	−2.61	−0.00
	Black/African	**<0.001**	−3.84	−1.48
	Latino	1.000	−2.16	0.95
	Arab	1.000	−1.23	1.02
Chinese	White/Caucasian	1.000	−1.71	3.88
	Indian	1.000	−3.17	2.73
	Black/African	1.000	−4.48	1.33
	Latino	1.000	−2.59	3.55
	Arab	1.000	−1.90	3.86
Indian	White/Caucasian	**0.048**	0.00	2.61
	Chinese	1.000	−2.73	3.17
	Black/African	0.141	−2.88	0.17
	Latino	1.000	−1.12	2.53
	Arab	0.282	−0.28	2.68
Black/African	White/Caucasian	**<0.001**	1.48	3.84
	Chinese	1.000	−1.33	4.48
	Indian	0.141	−0.17	2.88
	Latino	**0.008**	0.31	3.80
	Arab	**<0.001**	1.18	3.93
Latino	White/Caucasian	1.000	−0.95	2.16
	Chinese	1.000	−3.55	2.59
	Indian	1.000	−2.53	1.12
	Black/African	**0.008**	−3.80	−0.31
	Arab	1.000	−1.21	2.21
Arab	White/Caucasian	1.000	−1.02	1.23
	Chinese	1.000	−3.86	1.90
	Indian	0.282	−2.68	0.28
	Black/African	**<0.001**	−3.93	−1.18
	Latino	1.000	−2.21	1.21
